# Spontaneous Intramuscular Haematomas: A Single-Centre Retrospective Study and Proposed Management Algorithm

**DOI:** 10.7759/cureus.75327

**Published:** 2024-12-08

**Authors:** Malin Gunawardena, Abubaker Elamin

**Affiliations:** 1 General Surgery, Università degli Studi di Milano, Milan, ITA; 2 General Surgery, Humanitas University, Milan, ITA; 3 Otolaryngology, Nottingham University Hospitals, Nottingham, GBR

**Keywords:** acute abdominal surgery, iliopsoas hematoma, interventional radiology guided embolization, spontaneous rectus sheath hematoma, spontaneous retroperitoneal hematoma

## Abstract

Background: Spontaneous intramuscular haematomas by definition are haematomas without known etiology and exclude those caused by trauma, surgery, and muscular disease. This is a rare condition which has been increasing in incidence lately largely due to anticoagulant therapy use and currently, there is no level 1 evidence with regards to the best management of these patients, with different institutions using different approaches to treatment.

Materials and methods: We retrospectively analyzed 31 patients who were treated with the diagnosis of spontaneous intramuscular haematoma in our center, in the years between 2013 and 2017. Patient information was recorded including patient characteristics, vital parameters, biochemical data, imaging data, risk stratification scores, and patient outcomes.

Results: We observed that on average males had longer hospitalizations and a higher baseline platelet count was a protective factor against haemorrhage. On temporal evaluation of haemoglobin levels, we saw an important drop of haemoglobin at T6 for the transfused patients. The majority of patients (61.29%) were treated with arterial embolization, with great technical and clinical success (94% and 81% respectively). There was also a very strong association between patients treated with non-operative therapy (NOT) and shorter hospitalization.

Conclusion: NOT and embolization therapy are very effective treatments for spontaneous haematomas in the appropriate patient and should be included in the protocol. The small sample size was a very large limitation for the study, and a multicentric study is needed to further strengthen any associations we saw between risk factors and outcomes. We propose the need to perform a prospective study to ascertain the appropriate follow-up times for such investigations.

## Introduction

Background 

The spontaneous intramuscular hematoma of the abdomen, pelvis, and upper and lower limbs is a clinical condition characterized by haemorrhage without a known aetiology. By definition, this excludes iatrogenic, traumatic, or muscular disease causes and patients who have had surgery in the three weeks leading up to the event [1].

The first ever reported case of spontaneous abdominal bleeding in the literature was in 1909, by Maurice Barber, who observed spontaneous intra-abdominal bleeding in a 32-year-old woman, two days postpartum [2]. In 1911 a second case of spontaneous abdominal haemorrhage was reported by John Churchman [3]. After that, there have been sporadic mention of this condition, the rarity of which is elucidated by a literature review spanning 89 years (1909-1998) by Carmeci et al. [4] which gathered only 110 cases, and by Lafferty and Pearson who only found one case of abdominal apoplexy in 9560 autopsies performed over the course of 10 years [5].

This once-rare condition has been growing in incidence in recent years, largely due to a global increased anticoagulation therapy use by an ageing population [1,6]. The disconcerting aspect of this statistic is that haemorrhagic complications occur in many patients with coagulation profiles maintained within the appropriate therapeutic ranges [7]. In the acute setting, this constitutes a true emergency; as the initial presentation can range in severity from non-specific symptoms such as abdominal pain, hypotension and tachycardia to a more severe presentation such as an acute abdomen with hypovolemic shock; which poses an important diagnostic challenge to the attending physician [8,9]. Diagnostic delay and uncertainty can result in increased morbidity and mortality for this patient population, with nonsurgical mortality reaching as high as 100% [9].

At present clinicians are aided by modern radiological techniques which greatly help in reaching diagnosis and localization of the site of bleeding. Computed tomography (CT) is considered the gold standard for detecting spontaneous bleeding in stable patients, with sensitivity and specificity reaching 100% [10]. The management strategy mainly depends on the patient's haemodynamic conditions. This can range from a simple watch-and-wait approach in the stable patient to emergency laparotomy in the haemodynamically unstable or patients with high-volume hematomas, at risk of organ ischemia and compartment syndrome [11].

Growing trends in anticoagulation use are expected to increase the incidence of spontaneous hematomas and inexperienced clinicians should be given a clear management plan to follow in order to minimize time to diagnosis and treatment, ultimately reducing the associated morbidity and mortality.

## Materials and methods

Study aim

Spontaneous haematomas, being a potentially fatal and until recently rare clinical condition, need a clear and defined management protocol in order to facilitate diagnosis and treatment. Our study had the primary goal of developing a spontaneous haematoma management protocol encompassing NOT, endovascular embolisation, and surgical strategies which until now, to the best of our knowledge, has not been proposed. This was done through the evaluation of trends in risk factors amongst our study population.

Outcomes

To define a treatment protocol, we first wanted to check if any of the variables had an effect on patient outcomes. In our study the patient outcomes were taken as (1) the number of days the patient was admitted after the presentation of the haematoma; (2) complication rates; (3) transfusions needed.

Inclusion and exclusion criteria

All patients who were treated with the diagnosis of spontaneous haematomas, in the years between 2013 and 2017 were initially assessed for eligibility. All haematomas due to traumatic, iatrogenic, or other identifiable causes, did not fall into our definition of ‘’spontaneous haematoma’’ and thus were excluded from our study. All haematomas in line with our inclusion criteria were evaluated, except for those occurring in the head, neck, and spinal cord regions.

Study population

An ad hoc database was created for all the patients who were finally chosen for our study. The database consisted of 82 variables, which included patient details, and specific comorbidities of interest (such as diabetes mellitus, hypertension, coagulopathies, cardiopathies, thrombotic events, history of oncological disease, and rheumatological disease) as well as pharmacological therapy (including any anticoagulant, anti-platelet or steroids).

Additionally, vital parameters of blood pressure, heart rate, respiratory rate, and oxygen saturation on admission (Time 0 hours, or T0), biochemical data including haemoglobin, haematocrit, platelet count, international normalised ratio, activated partial thromboplastin time and lactate (at T0, T6, T12 and T24 post admission), and radiological data (the presence of venous or arterial blush and haematoma dimensions at T0 hours and T12 hours from admission) were also documented.

Therapeutic options such as NOT, embolisation, or surgical, and the number of transfusions (including the differential of concentrated blood products, fresh frozen plasma, or platelets), were also recorded.

We also documented haematoma locations, patient outcomes (including days recovered, therapeutic failure, and complications where applicable), and pre-discharge imaging of the haematoma.

Finally, we also used risk stratification tools to calculate patient morbidity/mortality and looked for any correlation with our patients' clinical severity and outcome. The risk stratification tools used in the study were: the American College of Surgeons (ACS) National Surgical Quality Improvement Program score [12], the Simplified Acute Physiology Score (SAPS II) [13], the Charlson Co-morbidity Index (CCI) [14], the Clinical Frailty Scale (CFS) [15] and the Barthel index [16].

Study flowchart

In total, we analyzed 208 patients who were diagnosed with haematomas in our centre; 177 were eliminated as they did not fit our inclusion criteria. The final study population comprised 31 patients.

**Figure 1:**
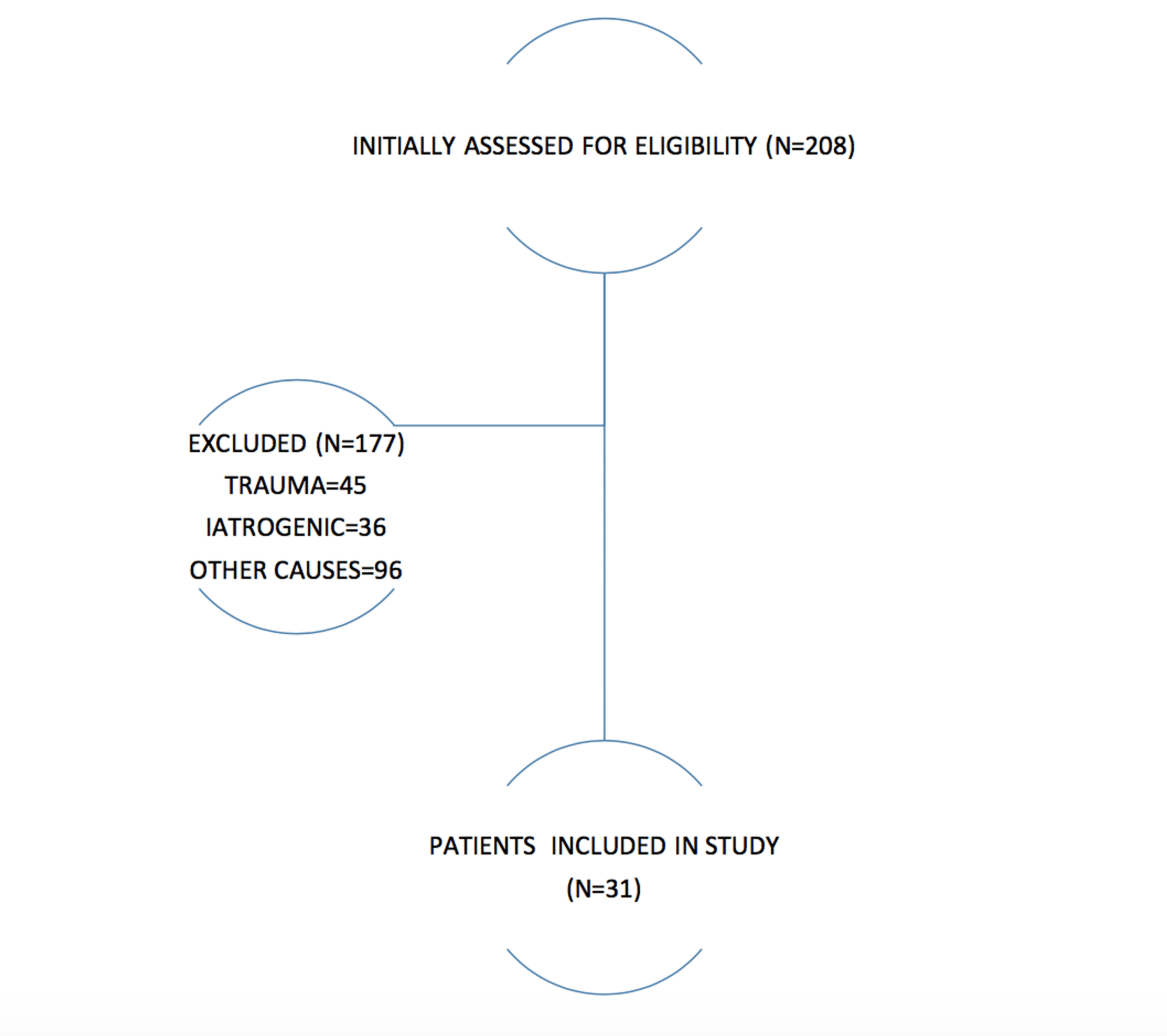
Study population

CT examination

A total of 28 patients (90.3%) had an urgent CT performed at T0. In our centre, we performed multidetector computed tomographic angiography (MDCTA) with a 16-detector CT scanner (Philips Ingenuity, Amsterdam, The Netherlands), and administered an intravenous injection of 120ml contrast material (Iomeron 350, Bracco Imaging, Milan, Italy). The scans were obtained in the basal, arterial (bolus-tracking technique was used with 50-60 second delay), and venous phases (180 seconds) with scanning parameters of 120 kVp, 200 mA, and 3-mm to 5-mm slice thickness. The haematoma volume was calculated by taking 2 diameters and using the formula of the ellipsoid: V = 4/3 * π * r3

Percutaneous transarterial embolisation

All trans-arterial embolisations were performed in an angiographic suite dedicated to interventional radiology (V3000; Philips Medical System, Amsterdam, The Netherlands) which was equipped with cone-beam CT (Siemens Artis-Zee, Munich, Germany). The materials used for arterial embolisation included: Glubran gel + lipiodol (liposoluble contrast), microparticles (400-500 micron diameter), and micro spirals. The choice of embolising agent is multifactorial having to take into account the location, size of the artery, and also operator confidence. The most efficacious is the Glubran glue, being used by more experienced operators, however, it has an increased level of difficulty when compared to the other options. Micro spirals are used for larger arteries whilst microparticles are used if we want to achieve temporary arrest of blood flow, especially in cases where we are not sure about the artery being embolised.

Statistical analysis

Data was described as numbers and percentages if categorical, or mean ± standard deviation (range) if continuous. Association among the outcome and the possible risk factors were explored with the appropriate regression analysis. For the days of hospitalisation due to spontaneous haematoma, we used linear regression, and results were expressed as a regression coefficient with a 95% confidence interval; the more the coefficient is distant from 0, the stronger the association. Regarding the need for transfusion and worsening of haemoglobin, we used logistic regression, and the results were expressed as an odds ratio (OR) with a 95% confidence interval; once again, the more the coefficient is distant from 1, the stronger the association is. All analysis was conducted using Stata 15 (StataCorp LLC, College Station, TX). A p-value under 0.05 was considered significant.

## Results

Results

Our final study population consisted of 31 patients, with a mean age of 76±12 years at presentation and an average length of stay under hospital admission of 13.48 ± 12.12 days. The majority of patients were cardiopathic (22 patients, 70.96%) and hypertensive (21 patients, 67.74%) with a quarter of the patients having deep vein thrombosis/pulmonary embolism (eight patients, 25.80%). Anticoagulants, anti-platelets or a combination of both drugs were taken by almost all our patients (29 patients, 93.5%), with only two out of the 31 (6.45%) being non-consumers (Table 1).

**Table 1:** Patient characteristics at baseline The data has been represented as N (%) and Mean±SD (%)* PE/DVT: deep vein thrombosis and pulmonary embolism; NOAC: novel oral anticoagulants; ACS NSQIP: American College of Surgeons National Surgical Quality Improvement Program

Variable	Value
Sex (M)	13 (41.94%)
Age*	76.32 ± 11.66 (49-95%)
BMI*	27.00 ± 6.89 (20.28 - 52.12%)
Hypertensive	21 (67.74%)
DM	5 (16.13%)
Coagulopathy	3 (9.68%)
PE/DVT	8 (25.80%)
History of cancer	10 (32.26%)
Cardiomyopathy	23 (74.19%)
Rheumatological disease	5 (16.13%)
Corticosteroid usage	12 (38.71%)
Anticoagulation/anti-platelet	29 (93.55%)
Aspirin	9 (29.03%)
Heparin	20 (64.52%)
Warfarin	10 (32.26%)
NOAC	2 (6.45%)
SAPS II*	31.58 ± 11.05 (12-54)
Clinical Frailty Score*	5.32 ± 1.51 (1-7)
ACS NSQIP*	3.13 ± 1.23 (1-5.5)
Barthel*	65.6 ± 28.95 (10-100)
Charlson*	4.83 (0-9)

**Table 2:** Biochemical data T0-T24 (hours) The data has been represented as N (%) and Mean±SD (%). The data has been represented as frequency (%) and mean ± standard deviations (%), respectively. Hb: haemoglobin; Hct: haematocrit; PLT: platelet count; INR: international normalised ratio; aPTT: activated partial thromboplastin time

Time periods	N	Mean ±SD (Range)
T0		
Hb	29 (93.5%)	9.02 ± 2.55 (4.7-13.9)
Hct	29 (93.5%)	28.37 ± 9.19 (14.8-43.5)
PLT	29 (93.5%)	250.10 ± 107.00 (21-456)
INR	22 (70.9%)	2.49 ± 3.40 (0.95 - 13.22)
aPTT	22 (70.9%)	1.30 ± 0.48 (0.77 - 2.59)
T6		
Hb	15 (48.3%)	8.89 ± 3.07 (4.2-14)
Hct	15 (48.3%)	27.24 ± 9.40 (13.7-43.9)
PLT	15 (48.3)	225.47 ± 84.80 (91-416)
INR	7 (22.5%)	1.38 ± 0.47 (0.95 - 2.06)
aPTT	6 (19.3%)	1.07 ± 0.19 (0.86 - 1.43)
T12		
Hb	17 (54.8%)	9.85 ± 1.77 (6.1 -13.2)
Hct	17 (54.8%)	28.32 ± 8.65 (2.7 - 40.6)
PLT	17 (54.8%)	196.65 ± 72.99 (82-389)
INR	11 (35.4%)	1.24 ± 0.33(1-2.2)
aPTT	10 (32.2%	1.13 ± 0.26 (0.9 - 1.8)
T24		
Hb	27 (87.0%)	9.19 ± 1.52 (6.4-12.6)
Hct	27 (87.0%)	28.01 ± 4.96 (19.8-39.6)
PLT	27 (87.0%)	210.81 ± 95.15 (20-412)
INR	8 (25.8%)	1.24 ± 0.36 (1-2.1)
aPTT	8 (25.8%)	1.01 ± 0.25 (0.8 - 1.6)

In our study, we collected biochemical data including Hb, Hct, PLT, INR, and aPTT levels at 0, 6, 12, and 24 hours since admission. The mean haemoglobin level at T0 was 9.02 ± 2.55 mg/dl with a range between (4.7-13.9), with a mean platelet count of 250.10 ± 107.00 (21-456) and an average INR of 2.49 ± 3.40 (0.95-13.22) at presentation. The initial radiological investigation was a CT scan for the majority of the patients (27 patients, 87.10%) which was double the amount of initial ultrasound scans performed (13 patients, 43.33%) (Table 3). Of our 31 patients, almost half of them had active bleeding in the form of an arterial or venous blush (13 patients, 48.15%). The maximum measurement of the haematoma volume had a mean value of 136.81 mm ± 78.13 (0-300), however, haematoma volumes could not be calculated for all the patients as some patients had incomplete records (Table 4).

**Table 3:** Imaging on admission The data has been represented as N and % for frequencies and percentages, respectively.

Variable (T0)	Frequency	Percentage
Ultrasound	13	43.33
CT	27	87.10
Arterial blush	5	19.23
Venous blush	9	34.62
Arterial or venous blush	13	48.15

**Table 4:** Haematoma characteristics The data has been represented as N (%), Mean±SD (range)

Variable (T0)	N (%)	Mean ± SD (range)
Length	20 (64.5%)	83.90 ± 56.56 (0-200)
Width	14 (45.1%)	52.21 ± 32.75 (0-95)
Height	19 (61.2%)	114.10 ± 89.06 (0-300)
Haematoma volume	21 (67.7%)	136.81 ± 78.13 (0-300)

Anticoagulant/antiplatelet use was not distributed evenly in our study population. There were four main classes of drugs that were taken into consideration; heparin (LMWH) was the most prescribed drug in this category, accounting for more than half of the prescriptions (n=20; 64.52%). Warfarin and aspirin were taken in similar proportions with 32.26% (n=10) and 29.03% (n=9) respective usage. The least used class of drugs in our study population were the novel oral anticoagulants which only accounted for 6.45% (n=2). Four patients (12.90%) required reversal of anticoagulation medication in the emergency department, two (6.45%) patients received prothrombin complex whilst the other two (6.45%) patients were infused with vitamin K. One (3.22%) patient refused blood transfusions on religious grounds.

**Figure 2:**
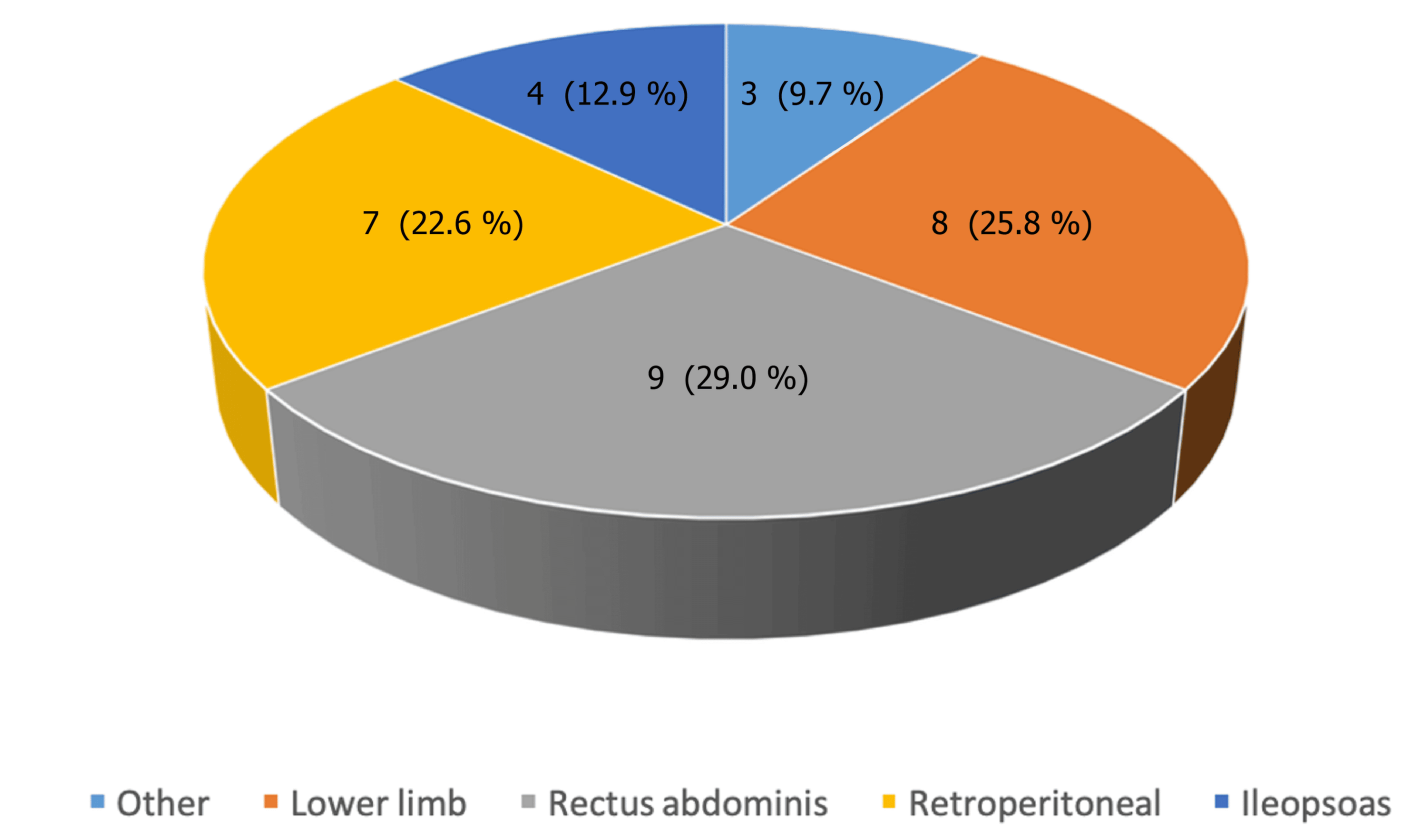
Spontaneous intramuscular haematoma distribution The data has been represented as N (%). A p-value of <0.05 is considered significant. There was no statistically significant association between haematoma location and days of hospitalisation (p = 0.5942) (Kruskal Wallis).

The spontaneous haematomas recorded showed varied anatomical distribution amongst our patients. The majority of the haematomas were found in the rectus abdominis muscle group (n=9; 29.03%) followed by spontaneous bleeding in the lower limbs (n=8; 25.81%), retroperitoneal space (n=7; 22.58%), iliopsoas muscle (n=4; 12.90%) and finally in the others category (n=3; 9.68%) (Figure 2). There is no association between haematoma location and days of hospitalisation that can be considered to be statistically significant (p = 0.5942 (Kruskal Wallis).

With regards to the therapeutic approach, there were three possible strategies: NOT, embolisation and surgical intervention. Where possible patients were treated with NOT (12 patients, 38.71%), however, the vast majority of the patients were treated through embolisation procedures (19 patients, 61.29%); of these 19 patients, two (10.53%) needed the surgical option. Therapeutic failure occurred in two patients who did not achieve complete haemostasis with the initial treatment. The mortality was 12.90% with three patient deaths resulting from complications whilst admitted.

Variables affecting the length of stay in the hospital

To evaluate the risk factors for longer hospitalisations, linear regression analysis was performed to look for associations between variables and the length of hospitalisation (days). The most distant from 0 coefficient is indicative of a higher association (Table 5).

**Table 5:** Association between variables and the length of hospital stay Linear regression analysis was performed with the most distant from 0 coefficient being indicative of a higher association. A p-value of <0.05 was considered significant. Hb: haemoglobin; PLT: platelet count; INR: international normalised ratio; aPTT: activated partial thromboplastin time; SAPS II: Simplified Acute Physiology Score II; NOT: non-operative therapy

Variable	Coefficient (95%CI)	P
Sex (M)	7.380(-1.365 to 16.126)	0.095
Age	0.117 (-0.275 to 0.510)	0.546
BMI	-0.380(-1.112 to 0.351)	0.294
Hb - T0	0.845(-1.041 to 2.731)	0.366
PLT - T0	-0.038(0.081 to 0.006)	0.085
INR - T0	-0.232(-1.471 to 1.007)	0.700
aPTT - T0	2.468(-6.351 to 11.286)	0.566
SAPS II	0.089(-0.327 to 0.504)	0.665
Barthel	0.107(-0.062 to 0.275)	0.203
Clinical Frailty Score	-0.309(-3.703 to 3.085)	0.852
NOT	-11.531(-19.739 to -3.322)	0.008

**Figure 3:**
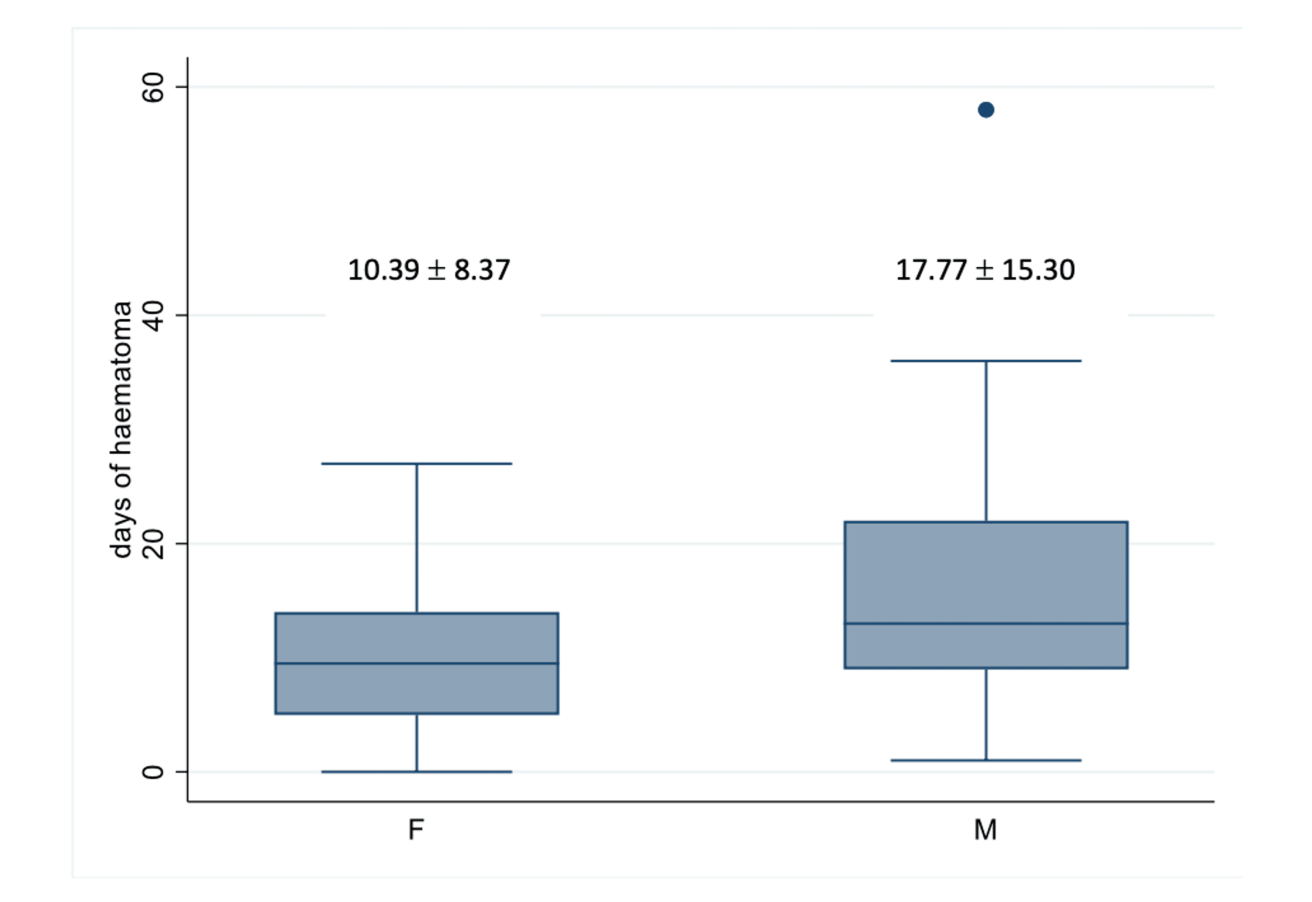
Length of hospital stay according to gender There were no significant statistical differences between male and female gender in terms of hospital stay (Wilcoxon p = 0.1926). A P-value of <0.05 is considered significant.

Patients treated with NOTs had the lowest length of hospital stay when compared with other treatment modalities. We noted male patients had longer hospital admissions when compared to their female counterparts, this, however, was not statistically significant (Wilcoxon p = 0.1926) (Figure 3).

**Figure 4:**
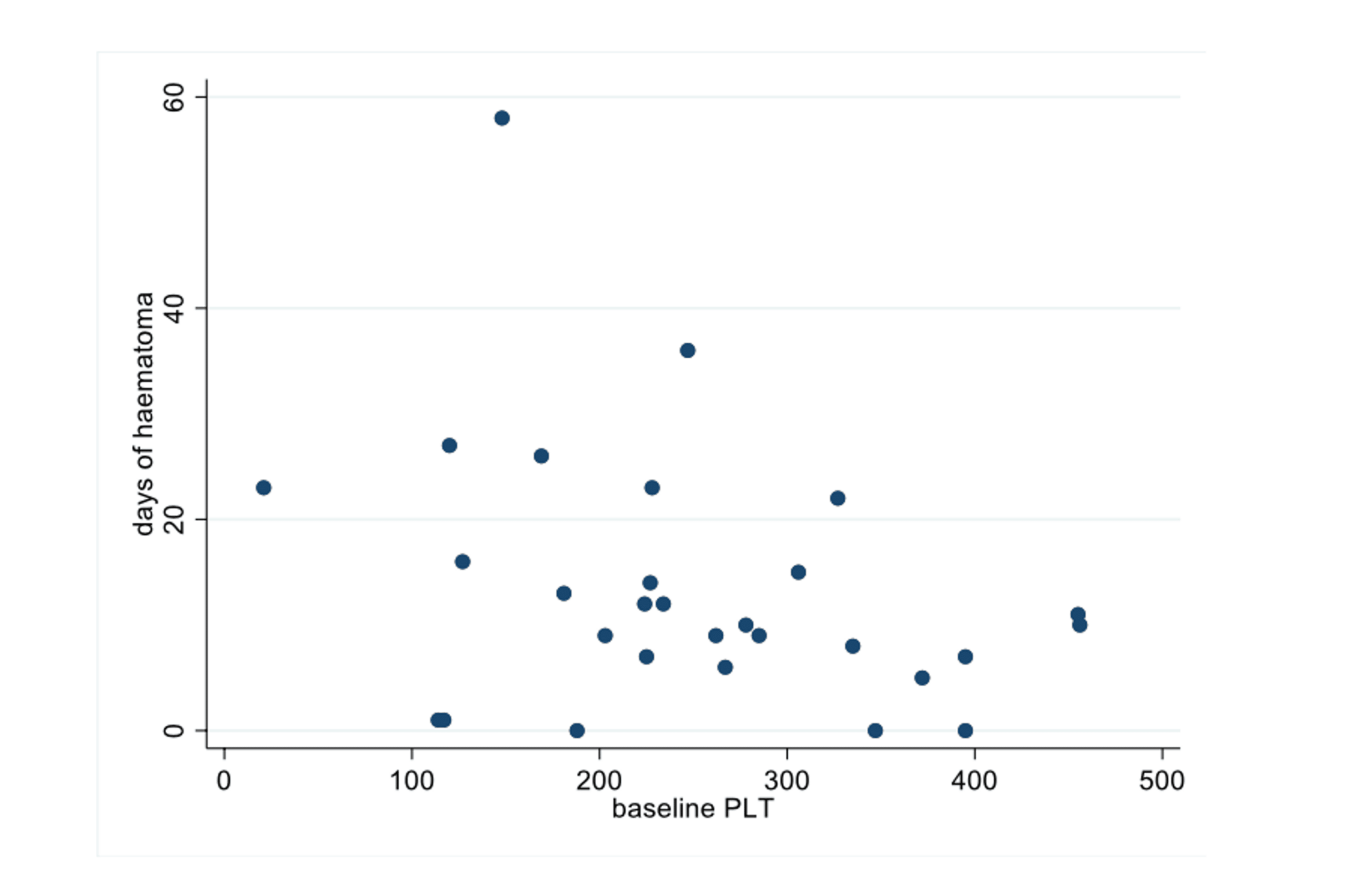
Platelet count at T0 No significant statistical difference between baseline platelet count (T0) and hospital stay (P= 0.085). P-value <0.05 considered significant)

There seems to be an inverse relationship between the baseline platelet count and the days of hospital admission. As shown in Figure 4, with an increase of the baseline platelet count there is a decrease in the number of days admitted to hospital. However, according to our analysis, this didn’t prove to be statistically significant.

Complication rate

**Figure 5:**
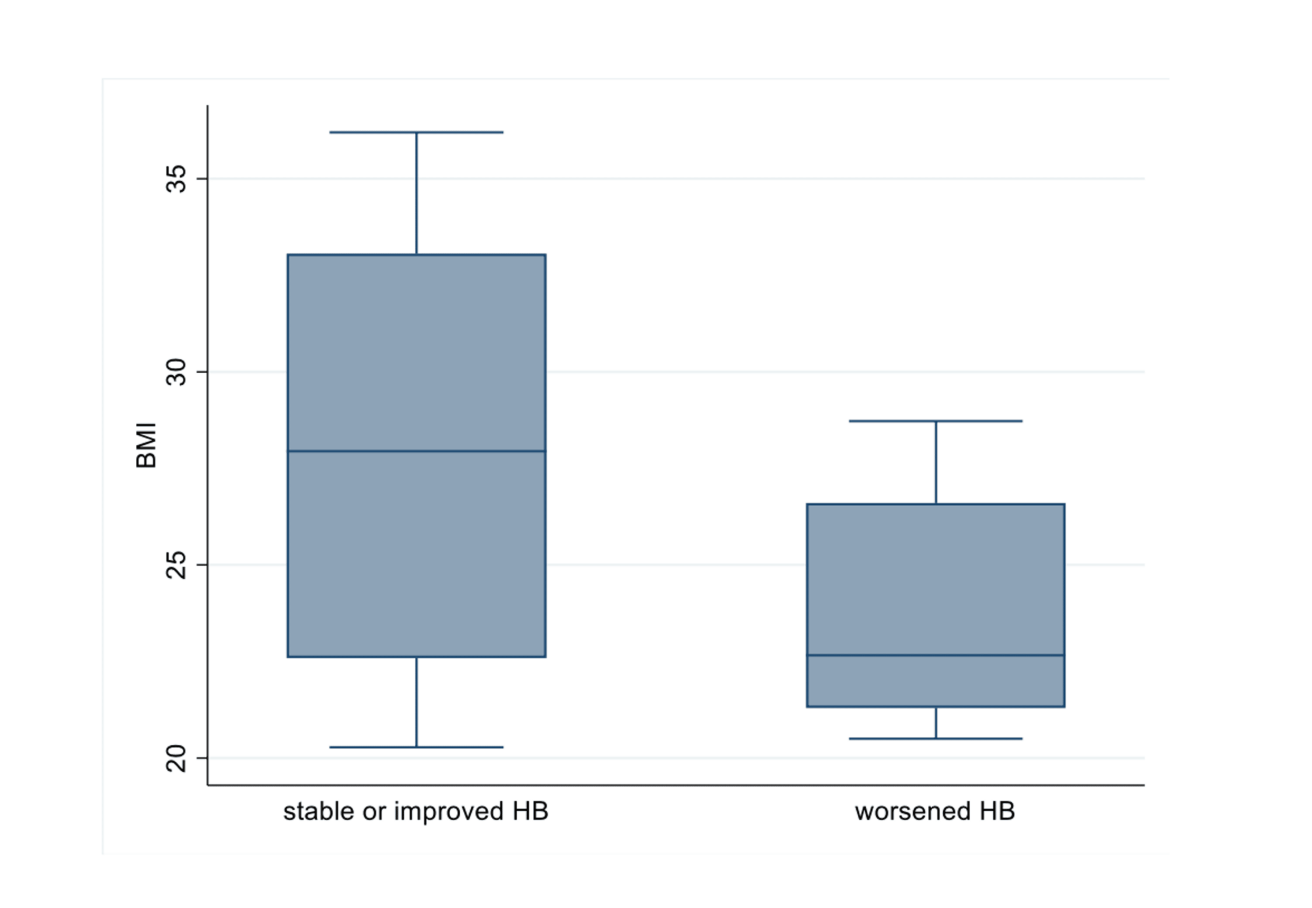
BMI vs worsening or stable/improving haemoglobin

We observed two complications in our study group; this was too small a number to look for any association so we looked for associations with the worsening of haemoglobin. The worsening of the haemoglobin was defined as a drop of at least 1 g/dl hb (if the change was within 1 g/dl we considered this as a stable hb level). We also noticed higher BMI to be a protective factor with regards to decreasing Hb with an odds ratio of 0.82 and P 0.077 (Table 6, Figure 5).

**Table 6:** Association between variables and worsening of haemoglobin Logistic regression with results expressed as an odds ratio (OR) with a 95% confidence interval; the more the coefficient is distant from 1, the stronger the association is. A p-value <0.05 is considered statistically significant. SAPS II: Simplified Acute Physiology Score II; NOT: non-operative therapy; Hb: haemoglobin; Hct: haemocrit; PLT: platelet count; INR: international normalised ratio; aPTT: activated partial thromboplastin time

Variable	OR	(95%CI)	P
Barthel	1.011	(0.983 to 1.040)	0.459
Clinical Frailty Score	0.724	(0.405 to 1.295)	0.277
SAPS II	1.018	(0.950 to 1.090)	0.622
NOT	0.926	(0.209 to 4.108)	0.919
Age	0.974	(0.908 to 1.044)	0.462
Sex (Male)	0.311	(0.068 to 1.427)	0.133
Hb - T0	1.239	(0.892 to 1.721)	0.201
Hct - T0	1.055	(0.955 to 1.166)	0.289
PLT - T0	0.998	(0.660 to 1.021)	0.632
INR - T0	0.883	(0.669 to 1.165)	0.378
aPTT- T0	0.337	(0.619 to 1.751)	0.880
Lactate	1.041	(0.673 to 1.897)	0.645
BMI	0.82	(0.66 to 1.02)	0.077

Need for transfusion

Associations between transfusions and our variables were investigated (Table 7). We noticed that those with lower haemoglobin or haematocrit levels, a higher SAPS II, and older patients had a higher probability of needing transfusions whilst a higher BMI had the opposite effect (Table 7).

**Figure 6:**
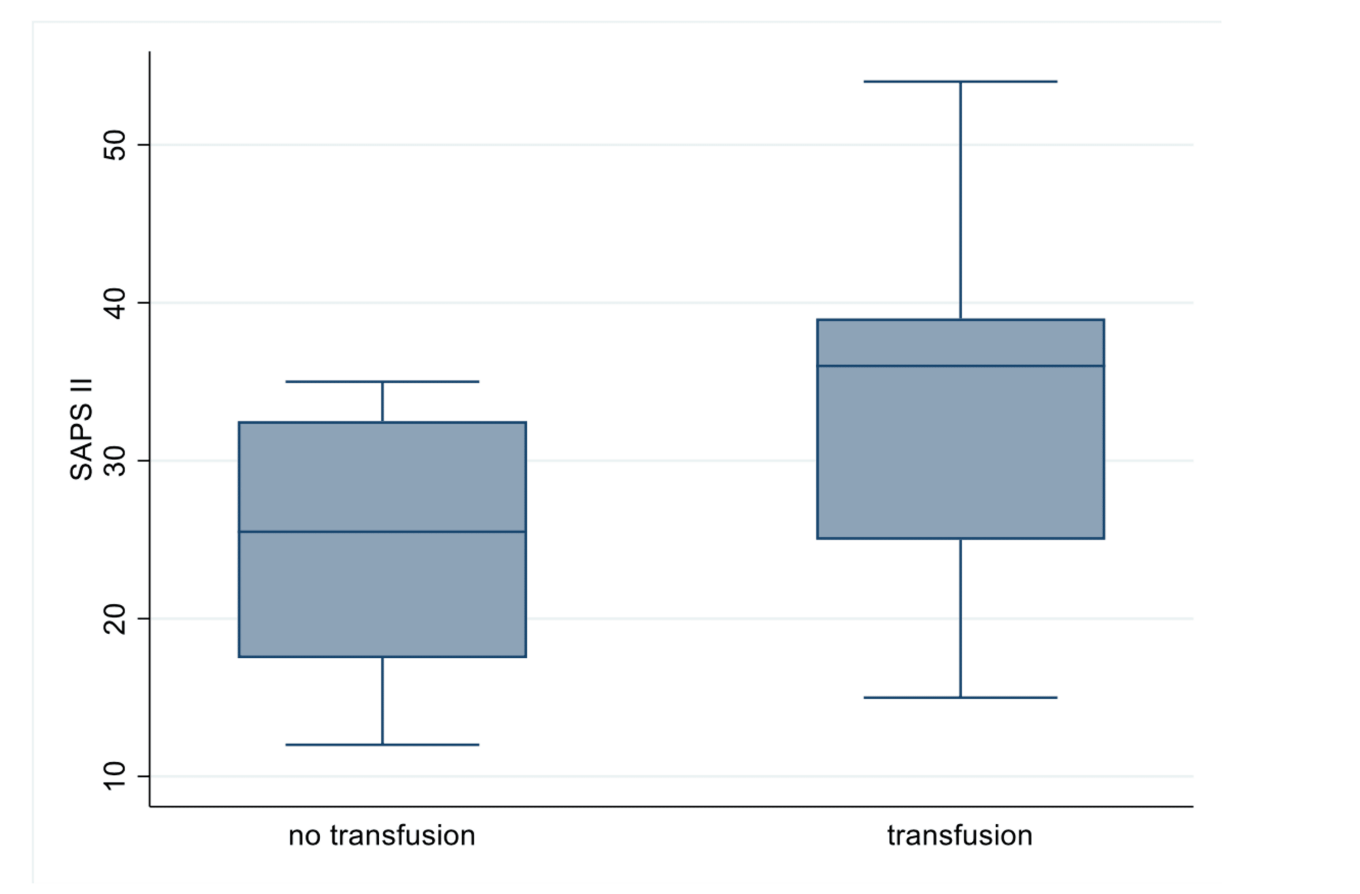
SAPS II for transfused vs non-transfused patients SAPS II: Simplified Acute Physiology Score II

**Table 7:** Association between our variables and need for transfusion Logistic regression with results expressed as an odds ratio (OR) with a 95% confidence interval; the more the coefficient is distant from 1, the stronger the association is. A p-value <0.05 is considered statistically significant. SAPS II: Simplified Acute Physiology Score II; NOT: non-operative therapy; Hb: haemoglobin; Hct: haemocrit; PLT: platelet count; INR: international normalised ratio; aPTT: activated partial thromboplastin time

Variable	OR	(95%CI)	P
Barthel	0.970	0.928 to 1.013	0.173
Clinical Frailty Score	1.447	0.783 to 2.674	0.239
SAPS II	1.093	0.999 to 1.197	0.052
NOT	0.533	0.104 to 2.722	0.450
Age	1.073	0.994 to 1.157	0.070
Sex (Male)	2.750	0.456 to 16.592	0.270
Hb - T0	0.435	0.247 to 0.767	0.004
Hct - T0	0.718	0.574 to 0.899	0.004
PLT - T0	0.999	0.992 to 1.007	0.918
INR - T0	1.376	0.642 to 2.950	0.412
aPTT - T0	0.703	0.107 to 4.632	0.715
BMI	0.840	0.692 to 1.019	0.077

We also looked at the haematoma locations and their associated SAPS II scores. There was no relation between the SAPS II and the haematoma location according to our analysis (Table 8).

**Table 8:** Mean SAPS II scores for each haematoma location SAPS II: Simplified Acute Physiology Score II

Location	N (%)	Mean ±SD(Range)
Other	3 (09.68%)	26± 11.31 (18-34)
Lower limb	8 (25.81%)	31.63±9.05 (16-41)
Psoas	4 (12.90%)	31.75±5.73 (25-39)
Retroperitoneal	7 (22.58%)	32.57±14.08(15-54)
Rectus abdominis	9 (29.03%)	31.44 ± (12-52)

**Figure 7:**
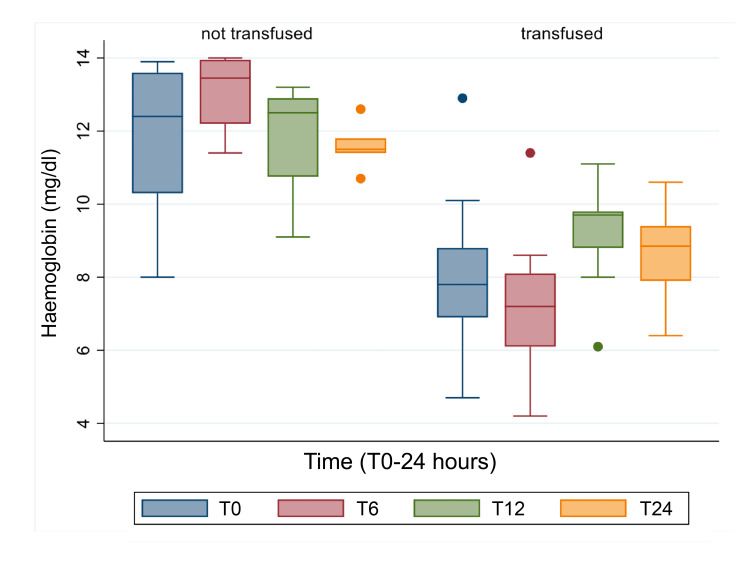
Comparison between haemoglobin levels between transfused and non-transfused patients at T0-T24

In the non-transfused patients, the Hb is relatively stable, whilst in the patients who needed transfusion we usually see a drop at T6 and this could be where you check (Figure 7). None of the other variables (location, drugs, clinical frailty score, Barthel, Charlson, max haematoma length) was associated with the need for transfusions according to our statistical analysis.

## Discussion

Although rare, spontaneous intramuscular haematomas are increasing in incidence nowadays, mainly due to anticoagulant therapy [8]. Worldwide epidemiological studies show us a growing trend in atrial fibrillation and DVT/PE prophylaxis so we are justified in claiming spontaneous intramuscular haematomas will likely become a common occurrence in emergency departments worldwide [17]. As such, awareness of the condition and a standardised management algorithm are extremely important in an effort to reduce associated morbidity and mortality.

One of the goals of our study was to detect risk factors for the development of spontaneous haematomas in an effort to identify at-risk patient groups. We performed regression analysis to look for associations between risk factors and our outcomes (days hospitalised, number of transfusions and worsening of the haemoglobin levels). Analysing the data we notice that male patients had overall longer hospitalisations and that the platelet count on admission (T0) was inversely proportional to the days of hospitalisation, with a higher initial platelet count being a protective factor. Although we could see possible associations between these risk factors and the length of hospitalisation, these were not proved to be statistically significant with p values of 0.095 and 0.085 respectively. This however is most likely attributable to the very small sample size of our study. The only statistically significant association was found to be between patients treated with NOT and the days of hospitalisation, with a p-value of 0.008 and a coefficient of -11.531 (-19.739 to -3.322). This is due to the fact that patients treated with NOT are less severe than those requiring embolisation or surgery. Surprisingly, patient age and haematoma location did not seem to affect the severity of the condition.

Patients with higher BMI seemed to have more stable haemoglobin levels, with fewer patients experiencing drops in their haemoglobin concentration over time. This finding mirrors similar claims by Wang et al. who state that BMI is a protective factor against blood loss and the need for transfusions [18]. They claim that procoagulant states characterised by increased levels of tissue factor, factor VIII, fibrinogen, inhibition of fibrinolytic pathways and greater platelet aggregability due to hyperlipidaemia and endothelial dysfunction of these patients are the reason for the findings [19]. This finding was incorporated into our treatment protocol since patients with lower BMI are at higher risk of worsening their condition and closer monitoring is required.

Taking SAPS II as an indication of severity for the group of patients, we noticed that our patient population had very similar levels of severity when compared to other large multi-centric studies. The 2019 Barral et al. study [20], which comprised almost four times as many patients as ours (n=112 patients), demonstrated almost identical SAPS II scores, with a mean score of 31.7 in their study in comparison to our mean of 31.6. In our study one of the focal points was the evaluation of biochemical data, looking at temporal trends across T0, T6, T12, and T24 (hours). Analysing this information, we notice that between T0 and T6, the haemoglobin levels remain relatively stable, increasing in concentration at T12 after which a slight reduction occurs at T24. The reason for the increasing haemoglobin levels between T6 and T12 is likely due to resuscitative efforts and blood transfusions provided by the attending medics, which would then normalise in the 12 hours following, as seen by the slight drop at T24.

Radiological information was another important area of interest in our study, with all patients initially investigated either by computed tomography or by an ultrasound scan. Volumetric calculation of the haematoma size was not possible in the majority of the cases as usually only one or two of the three measurements were recorded by the radiologists. It was also noted that follow-up imaging times were not consistent amongst our patients, with only a few repeat CT scans at T12 (n=5), T24 (n=2), and T48 (n=1) taken. Due to the fact that haematoma volume could not be calculated, we used the maximum measurement of any one of length, height or width to perform our statistical analysis. Interestingly we could not find any significant correlation between haematoma maximum dimension and our patient outcomes (days of hospitalisation, number of transfusions or worsening of haemoglobin levels over time)

With regards to treatment modality, there were three variables: NOTs, trans-arterial embolisation, and surgery. Generally, stable patients with no active bleeding were treated conservatively, whilst those who presented with an active arterial blush underwent embolisation procedures. This was found to be very much in line with recommendations put forth by Popov et al. [1] where they propose indications on trans-arterial embolisation vs NOT for patients. In our centre, the patients treated with embolisation had a 94% technical success rate and 81% clinical success rate; this proved to be in agreement with the figures reported in the large multicentric study performed by Barral et al. [20]. Additionally, the high technical and clinical success rates observed in our experience, echo the hypothesis proposed by Dohan et al., i.e. that embolisation is a safe and effective strategy in dealing with spontaneous haematomas [21]. Attesting to the notion that nowadays, surgery has become the second line when dealing with these uncomplicated patients, is the fact that in our study, surgery was only performed in two instances. The first was due to the therapeutic failure of an initial trans-arterial embolisation. The second instance was a haematoma incision and drainage performed by our vascular surgeons. NOT was 100% successful in our patients, with none of them experiencing therapeutic failure. In our centre the indications for a NOT were haemodynamic stability and no active bleeding on CT. The 100% success rate achieved is a good indication for the implementation of these parameters in our proposed treatment protocol.

When looking at the temporal variations of haemoglobin for both the transfused and the non-transfused patients, we find a very interesting observation. Whilst for the non-transfused patients we see that the haemoglobin remains relatively constant, we notice that there is a drop in haemoglobin concentration at T6 for the patients who were transfused. This could be an important recommendation to always control the blood haemoglobin levels at T6 in patients who need transfusions, as a drop is likely. To our knowledge, this information has not been stated anywhere in the literature prior to our study. After an extensive evaluation of all the risk factors and their potential correlations with our outcomes, we did not yield any additional associations.

Treatment flowchart

Our retrospective study highlighted the need for a universal treatment protocol, as we did not often see a congruency between the management of different patients. Depending on various factors, the patients presenting with spontaneous haematomas can be either overtreated or undertreated; this significant problem shows us there is a need for standardisation. With the information gathered in our retrospective study as well as consensus from multidisciplinary meetings between the various departments at our institution (emergency medicine, haematology, internal medicine, diagnostic radiology, interventional radiology, and general surgery), a Frailty index and proposed management algorithm were developed in a bid to solve this issue. This algorithm will now be used in our institution as well as other hospitals across Milan as part of a large multi-centric prospective clinical trial to prove its validity.

**Figure 8:**
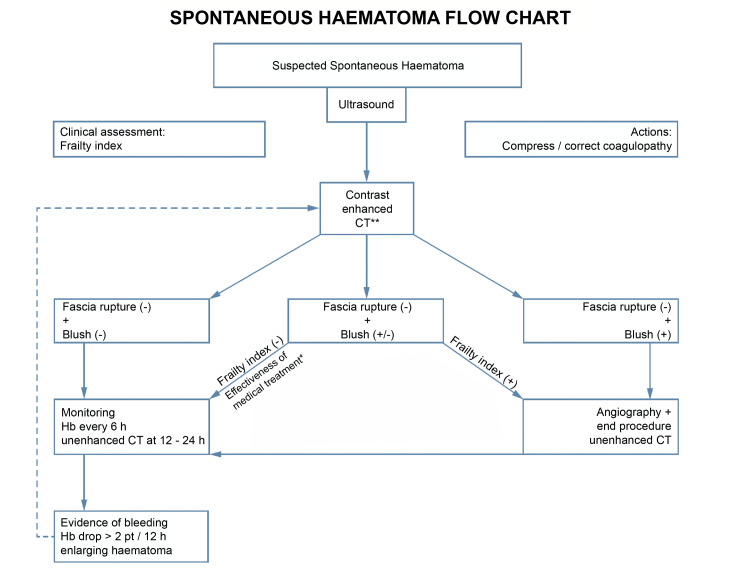
Proposed management algorithm for spontaneous haematoma * To be assessed together with on-call haematologist ** Unstable patients or patients with fascial rupture on CT scan require emergency surgery

**Table 9:** Frailty index If the patient had two or more of the above, they were deemed to have a high frailty index and at a high risk for deterioration. PRBC: packed red blood cells; COPD: chronic obstructive pulmonary disease; DM: diabetes mellitus

Component	Criteria
Age	> 70
Hb	< 9 or drop > 2g/dl in 4h, or more than 2 PRBC transfused
BP	< 90/60 (at presentation)
HR	> 110 (at presentation)
Haemostasis	Not correctable haemostasis defect
Comorbidities	>2 major comorbidities (eg: cardiopathy, COPD, DM, neoplasm, stroke)

Study limitations

The rarity of spontaneous haematomas was a significant limitation in this study. Being a single-centre retrospective analysis, our study population was limited to only 31 patients. This made subtle associations, although visible, not significant in our statistical workup. There was also major confounding with regard to days of hospitalisation in some patients since they either were already admitted to our hospital or developed complications that would ultimately lengthen their hospital stay. Increasing the patient number by involving other tertiary centres, and turning this into a multi-centric study would both increase the statistical power of our associations and enable us to reduce the effects of confounding. The practice of measuring intra-compartment pressure at the site of haematomas needs to further be encouraged. In our study, there was only one patient with reported intra-abdominal pressure measurements. This was done by measuring the bladder pressure using a Foley catheter, but only after the patient developed symptoms of compartment syndrome due to a retroperitoneal haematoma.

## Conclusions

To date, the absence of a standard management plan for patients with suspected spontaneous haematomas is problematic, especially considering the increasing number of reported cases around the world. Our study, however, yielded some important information which could be incorporated into the development of a universal management plan. The effectiveness of NOT for haemodynamically stable patients and embolisation for patients presenting with active bleeding is once again elucidated in our findings. We also found lower BMI, initial platelet counts and female sex all seem to be factors resulting in worse outcomes and they should be considered when assessing patients. In our investigation, we noticed an important haemoglobin drop in patients who required transfusions, so an important recommendation in our treatment protocol would be to request complete blood counts at T6 in all transfused patients. While some indications of the management and detection of spontaneous haematomas were seen in our study, the small sample size greatly reduced the significance of our findings. When taking into consideration the rarity of the investigated condition, involving other tertiary centres and performing a multi-centre study would be ideal as this would enable us to analyse a larger number of cases, thus significantly increasing the statistical power of our findings. Additionally, we propose the need to perform a prospective multi-centre study, with the implementation of our treatment algorithm and with appropriate follow-up times to be investigated as this would prove immensely useful in the management of these particular patients.
